# Anemia and iron homeostasis in a cohort of HIV-infected patients in Indonesia

**DOI:** 10.1186/1471-2334-11-213

**Published:** 2011-08-09

**Authors:** Rudi Wisaksana, Rachmat Sumantri, Agnes R Indrati, Aleta Zwitser, Hadi Jusuf, Quirijn de Mast, Reinout van Crevel, Andre van der Ven

**Affiliations:** 1 Department of Internal Medicine Faculty of Medicine, Padjadjaran University/Hasan Sadikin Hospital, Bandung, Indonesia; 2 Health Research Unit, Faculty of Medicine, Padjadjaran University, Bandung, Indonesia; 3 Clinical Pathology Faculty of Medicine, Padjadjaran University/Hasan Sadikin Hospital, Bandung, Indonesia; 4 Department of Internal Medicine, Radboud University Nijmegen Medical Centre, the Netherlands

**Keywords:** anemia, iron, HIV

## Abstract

**Background:**

Anemia is a common clinical finding in HIV-infected patients and iron deficiency or redistribution may contribute to the development of low hemoglobin levels. Iron overload is associated with a poor prognosis in HIV and Hepatitis C virus infections. Iron redistribution may be caused by inflammation but possibly also by hepatitis C co-infection. We examined the prevalence of anemia and its relation to mortality in a cohort of HIV patients in a setting where injecting drug use (IDU) is a main mode of HIV transmission, and measured serum ferritin and sTfR, in relation to anemia, inflammation, stage of HIV disease, ART and HCV infection.

**Methods:**

Patient characteristics, ART history and iron parameters were recorded from adult HIV patients presenting between September 2007 and August 2009 in the referral hospital for West Java, Indonesia. Kaplan-Meier estimates and Cox's regression were used to assess factors affecting survival. Logistic regression was used to identity parameters associated with high ferritin concentrations.

**Results:**

Anemia was found in 49.6% of 611 ART-naïve patients, with mild (Hb 10.5 - 12.99 g/dL for men; and 10.5 - 11.99 g/dL for women) anemia in 62.0%, and moderate to severe anemia (Hb < 10.5 g/dL) in 38.0%. Anemia remained an independent factor associated with death, also after adjustment for CD4 count and ART (p = 0.008). Seroprevalence of HCV did not differ in patients with (56.9%) or without anemia (59.6%). Serum ferritin concentrations were elevated, especially in patients with anemia (p = 0.07) and/or low CD4 counts (p < 0.001), and were not related to hsCRP or HCV infection. Soluble TfR concentrations were low and not related to Hb, CD4, hsCRP or ART.

**Conclusion:**

HIV-associated anemia is common among HIV-infected patients in Indonesia and strongly related to mortality. High ferritin with low sTfR levels suggest that iron redistribution and low erythropoietic activity, rather than iron deficiency, contribute to anemia. Serum ferritin and sTfR should be used cautiously to assess iron status in patients with advanced HIV infection.

## Background

Anemia is a common clinical finding in HIV-infected patients and is associated with advanced disease, lower quality of life and higher mortality [1-4]. Many factors may contribute to the development of anemia in HIV-infected patients including nutritional deficiencies, opportunistic infections, AIDS-related malignancies, drug treatment and a direct effect of HIV on the bone marrow [4]. Iron deficiency and inflammation-induced iron maldistribution may also contribute to HIV-associated anemia [5,6]. Due to the effects of inflammation, iron is diverted from the circulation into the reticulo-endothelial system and other storage sites. Apart from inflammation, also HCV may possibly contribute to redistribution of iron [7]. Hepcidin plays an important role in these processes [8,9], by limiting the availability of iron for hematopoiesis [10]. Iron maldistribution may have another unwanted effect; it may increase susceptibility to opportunistic infections, and accelerate disease progression [7,11-14]. Indeed, iron overload is associated with a poor prognosis in HIV and Hepatitis C virus infections [7].

Serum concentrations of ferritin and soluble transferrin receptor (sTfR) are frequently used to assess iron status [15]. Low ferritin is an indicator of iron deficiency, but as ferritin is also an acute phase reactant its interpretation is difficult in the presence of inflammation. Levels of sTfR are predominantly determined by the erythropoietic activity. Iron deficiency leads to increased erythroblast numbers and increased TfR expression and thus to considerably elevated sTfR levels. In contrast, anemia of inflammation is characterized by normal sTfR levels [6,16].

So far, limited and sometimes contradictory reports have been published on ferritin and sTfR in HIV-infected patients. High plasma ferritin concentrations have been found among HIV-infected patients [14,17-19], while other studies have reported low ferritin concentrations [20,21]. Co-infection with hepatitis C virus (HCV) may further complicate the assessment of iron status, as HCV infection is associated with high plasma ferritin concentrations [22]. With respect to sTfR levels in HIV patients, two studies found sTfR within the normal range [14,18], suggesting that sTfR is not affected by HIV infection [12]. However, this is in contrast with two other studies showing an increase in sTfR concentrations after initiation of antiretroviral treatment (ART) [19,21].

In the present study, we examined the prevalence of anemia and its relation to mortality in a cohort of HIV patients in a setting where injecting drug use (IDU) is the main mode of HIV and HCV transmission [23]. We also measured serum ferritin and sTfR, in relation to anemia, inflammation, stage of HIV disease, ART and HCV infection.

## Methods

### Setting and design

This cohort study was conducted at Hasan Sadikin hospital as the referral hospital for HIV in West Java, Indonesia. As one of the first 25 hospitals selected by the Indonesian government to provide HIV-care, Hasan Sadikin hospital has delivered free antiretroviral treatment and PCP-prophylaxis since December 2004. Following WHO and national guidelines [24,25], ART is indicated for patients presenting with WHO clinical stage IV, WHO clinical stage III with a CD4 count below 350/mm^3^, or WHO clinical stage I or II with a CD4 count below 200/mm^3^. The national program provides a choice of nevirapine (NVP), efavirenz (EFV), zidovudine (ZDV), stavudine (d4T) and lamivudine (3TC) as first-line ART. For this study, we included all HIV-positive patients above 14 years old presenting between September 2007 and August 2009. All patients signed informed consent and the study was approved by the hospital ethical committee.

### Data collection and laboratory examination

At time of first presentation at the hospital, all patients (both ART-naïve and ART-experienced) have a 'baseline visit' for structured interviewed and laboratory examination. Afterwards they come for scheduled visits, once-monthly when on ART, three- to six-monthly when not on ART. ART naïve patients were compared with patients with a favorable effect of ART, excluding those that with less than three months of ART, those who had ever interrupted ART for more than one month, those who had detectable HIV-RNA after six months ART, and those taking 2^nd ^ART. At presentation, data collected included gender, age, history of injecting drug use, body mass index (BMI, kg/m^2^), WHO stage, oral candidiasis, and history of ART or tuberculosis (TB) treatment. For ART-experienced patients, treatment duration and regimen were recorded. During follow-up, death was recorded from medical records.

Laboratory examinations included hemoglobin, red cell indices (Cell Dyne 3000, Abbot) and manual reticulocyte counts. Reticulocyte index was defined as the reticulocyte percentage × (measured hematocrit/normal hematocrit) [26]. Anti HCV antibodies were detected by an electrochemiluminescence assay, ECLIA (Elecsys 2010, Roche) and CD4-cell count by flowcytometry (BD Biosciences, Jakarta, Indonesia). In our setting, HIV-RNA (real time PCR, Abbott, USA) is only measured in patients taking ART, but not in ART naïve patients. Between January 2008 and June 2008 we also measured plasma ferritin (ECLIA method Elecsys 2010, Roche; reference range 30-400 ng/mL for men and 35-150 ng/mL for women), soluble Transferin Receptor (sTfR) (enzyme Immunoassay method, Diamed, Eurogen; reference range 1870-2450 U/mL) and high sensitive C-Reactive Protein (hsCRP) (immunoturbidimetry method, Hitachi 912, Roche; reference < 5 mg/L) in newly diagnosed patients. We defined ferritin as 'high' if it was above the upper reference value.

### Data analysis and statistics

WHO/ACTG criteria were used to define mild (Hb 10.5 - 12.99 g/dL for men; and 10.5 - 11.99 g/dL for women), moderate (Hb 8.0 - 10.49 g/dL) and severe (Hb < 8.0 g/dL) anemia [27,28]. As diagnosis of TB is difficult in HIV-positive patients in this setting due to its paucibacillary nature and the fact that many patients are unable to expectorate sputum [29], we considered all patients receiving TB treatment within three months after presentation as having TB co-infection. Mortality data were derived from clinical files, reports from community organizations or phone interviews from the clinic. Patients not returning for more than three months without confirmation of death or transfer were considered lost to follow-up. Data are presented as proportions or median with inter quartile range if not normally distributed. Categorical and continuous data were compared using the Chi-squared and Kruskal-Wallis tests respectively. Progression to death was measured by Kaplan-Meier estimates with Cox-regression to examine factors in baseline visits that affecting survival. Ferritin and sTfR concentration divided based on manufacturers reference values. Cox-proportional hazard regression model was used to investigate association between iron inflammatory parameters and mortality. Clinical parameters were examined as a possible determinant of mortality and high ferritin concentrations using logistic regression models. Variables which were significantly associated in univariate analysis (p < 0.05) were used in the final backward stepwise multivariate regression model. Proportionality was first assessed by generating time dependent covariates of interactions between predictors and survival function. Log-transformed ferritin and sTfR values were used to examine the relation between ferritin, sTfR, CRP and CD4. All statistical analysis was done using SPSS version 16.0.2 and Prism 4 for Windows.

## Results

### Patient characteristics

**Figure 1:**
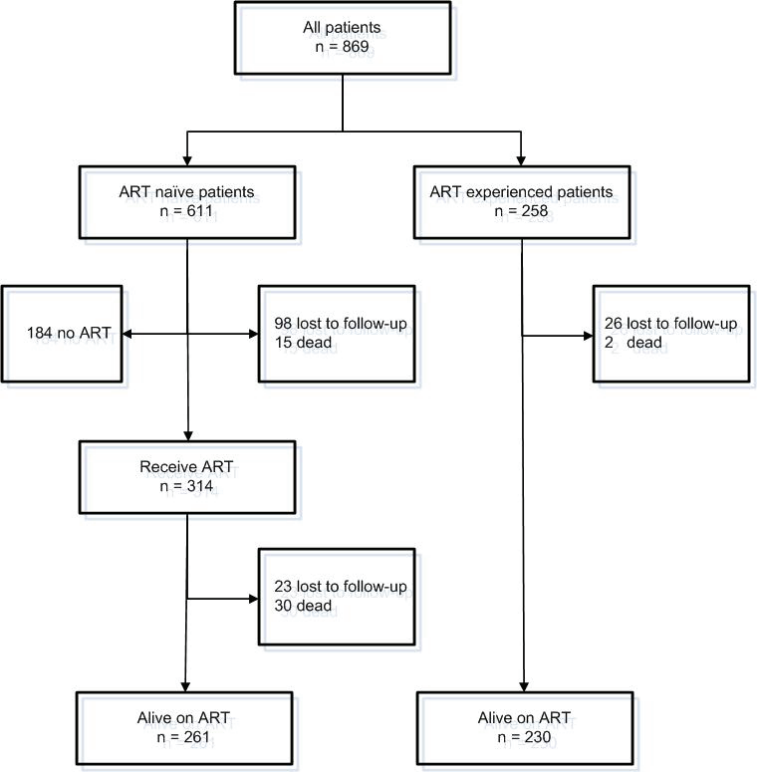
**Follow-up diagram of the cohort HIV-patients in Hasan Sadikin Hospital Bandung, September 2007-August 2009 (n = 869)**.

A total number of 869 HIV positive patients were enrolled in this study, 70.3% (611) of whom were ART naïve, and 258 (29.7%) of whom were on ART (Figure 1). Table 1 shows the baseline patient characteristics.

**Table 1:** Characteristics of ART-naïve and experienced HIV-patients in Hasan Sadikin Hospital, September 2007 - August 2009 (n = 869)

Characteristics	ART naïve				ART experienced				p value
	
	Total n = 611	No anemia n = 304	Mild n = 186	Moderate-severe n = 114	Total n = 258	No anemia n = 216	Mild n = 37	Moderate-severe n = 5	
Male sex, %	68.2	67.1	68.8	69.3	81.6	83.6	73.0	60.0	< 0.001
Median age, yrs (IQR)	28 (26-31)	28 (25-30)	29 (26-32)	29 (26-34)	29 (26-32)	29 (26-32)	30 (26-34)	31 (29-31)	0.45
Median BMI, kg/m^2^ (IQR)	19.5 (17.3-22.0)	19.8 (17.8-22.6)	19.1 (16.9-21.6)	19.6 (16.8-21.8)	20.3 (18.6-22.5)	20.4 (18.5-22.5)	20.2 (18.5-22.3)	17.8 (15.7-22.1)	0.63
History of IDU, %	63.1	64.9	61.8	61.0	84.4	86.5	78.4	40.0	< 0.001
Anti HCV (+), %	58.5	59.6	61.3	50.0	80.0	84.0	64.7	25.0	< 0.001
Median CD4, cells/mm^3^ (IQR)	76 (20-305)	232 (66-406)	45 (15-144)	17 (6-45)	272 (201-395)	280 (220-425)	195 (128-314)	190 (72-375)	< 0.001
TB treatment, %	21.3	6.3	27.4	50.9	3.9	2.8	5.4	40.0	< 0.001
Oral Candidiasis, %	27.8	21.8	35.4	31.4	3.3	2.0	5.7	40.0	< 0.001
Median MCV (IQR) (fl)	84.3 (80.3-87.5)	85.8 (82.8-88.6)	82.8 (77.6-86.4)	81.3 (76.0-84.7)	108.5 (103.0-114.0)	109.0 (104.0-114.0)	108.0 (99.8-114.0)	88.4 (81.0-100.1)	< 0.001
Median MCH (IQR) (pg)	28.9 (27.3-30.2)	29.5 (28.2-30.5)	28.4 (25.9-29.8)	27.8 (25.7-29.2)	38.4 (36.0-40.6)	38.4 36.3-40.7)	39.0 (34.2-40.5)	29.2 (27.7-33.8)	0.58
Reticulocyte index (%)	0.8 (0.6-1.5)	1.0 (0.6-1.8)	0.8 (0.6-1.0)	1.0 (0.5-2.7)	0.8 (0.6-1.0)	0.9 (0.7-1.4)	0.6 (0.5-1.0)	1.0 (1.0-1.0)	0.02

* p value for difference between ART-naïve and experienced group

Mild anemia: Hb 10.5 - 12.99 g/dL for men; and 10.5 - 11.99 g/dL for women, moderate-severe anemia: Hb < 10.5 g/dL.

TB treatment: receive TB treatment in presentation or within 3 months of presentation

Anemia status missing in 7 patients, gender in 3 patients, age in 1 patients, BMI in 151 patients, IDU status in 46 patients, anti HCV in 117 patients, CD4 in 13 patients, oral candidiasis in 94 patients, reticulocyte index only available from 184 patients

BMI: body mass index; IDU: injecting drug user; HCV: Hepatitis C virus; TB: tuberculosis; MCV: mean corpuscular volume; MCH: mean corpuscular hemoglobin; IQR: interquartile range.

### ART naïve patients

In this group, mild, moderate and severe anemia was present in 30.4%, 14.1%, and 4.6%, respectively, while hemoglobin levels were missing for 7 patients (1.1%). Clinical characteristics of ART-naive patients, stratified according to anemia category are presented in Table 1. Most ART-naive patients presented with advanced HIV infection or AIDS; 66.7% had a CD4-cell count below 200 cells/mm^3^, and 62.8% were in WHO stage III/IV. Malnourishment, defined as a body mass index (BMI) below 18.5 kg/m^2 ^was found in 38.0% of patients; severe malnourishment (BMI < 16 kg/m^2^) was found in 12.7% of patients. One fifth (21.3%) of patients were already on TB treatment or started TB treatment within 3 months of presentation, 58.5% were co-infected with HCV, and oral candidiasis was diagnosed in 27.8% of patients.

Anemia in ART-naïve HIV patients was mostly normocytic, normochromic and characterized by a normal or low reticulocyte index suggesting 'anemia of chronic disease' as the main cause. Anemia was associated with a low CD4-cell count (p < 0.001), and TB treatment (p < 0.001) (Table 1). Hepatitis C co-infection prevalence was not different in patients with and without anemia (56.9% vs. 59.6%, p = 0.54). During follow-up, 314 patients (51.4%) started ART after a median of 28 days (IQR: 19-55). The majority of patients (70.1%), none of whom had moderate or severe anemia, were started on a ZDV-containing regimen. From this group, 13.6% developed anemia during follow-up and were subsequently switched from ZDV to d4T.

### Mortality in ART-naïve patients

**Figure 2:**
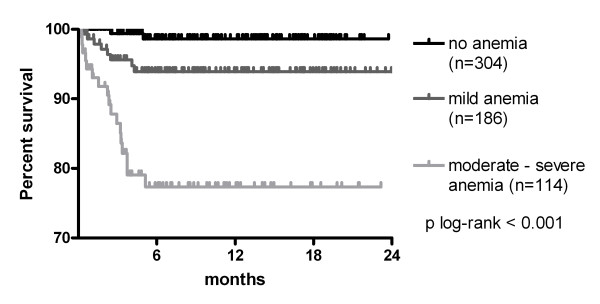
**Kaplan-Meier survival curve for ART-naïve HIV-infected patients with no (black), mild (dark grey) or moderate/severe anemia (light grey) (n = 604)**.

Anemia was associated with increased mortality during follow-up. When 611 ART-naïve patients were followed for a median of 4.4 (IQR: 0.0-12.1) months (total follow-up: 335 person-years), 45 patients (7.4%, IQR: 5.3%-9.5%) died and 121 (19.8%, IQR: 16.6%-23.0%) were lost to follow-up (Figure 1). Mortality (75.6% of the total) and loss to follow-up (92.6% of the total) were highest in the first 6 months after presentation. Using Kaplan-Meier estimates, the survival rates at 6 months were 98.6% (95% CI: 97.3-99.9%) for patients without anemia, 95.1% (92.0-98.5%) for patients with mild anemia and 82.5% (75.5-89.4%) for patients with moderate or severe anemia (*P *< 0.001; Figure 2). Beside a CD4 cell count below 50/mm^3 ^(HR: 5.7, IQR: 1.6-17.8, p = 0.003), moderate to severe anemia remained independently associated with an increased risk of death in multivariate analysis (HR: 6.5, IQR: 2.0-21.2, p = 0.002) (Table 2), This association remained significant after eliminating subjects with tuberculosis co-infection, which is an important risk factor for death.

**Table 2:** Multivariate analysis for factors related to six-month mortality among ART-naïve patients (n = 611) in Hasan Sadikin Hospital, September 2007 - August 2009

			Base Analysis				Sensitivity Analysis	
			
	n	Mortality	Univariate		Multivariate		Multivariate	
			
			HR (95% CI)	p value	HR adjusted (95% CI)	p value	HR adjusted (95% CI)	p value
Gender								
Female	194	5	1		1		1	
Male	416	29	2.8 (1.1-7.4)	0.03	1.5 (0.5-4.3)	0.46	1.8 (0.5-6.2)	0.32
Age (years old)								
< 29	317	11	1		1		1	
≥29	294	23	2.3 (1.1-4.9)	0.02	1.6 (0.7-3.5)	0.24	1.4 (0.5-6.2)	0.49
CD4 (cells/mL)								
> 50	353	4	1		1		1	
≤ 50	248	29	11.6 (4.0-33.3)	< 0.001	5.7 (1.8-17.8)	0.003	6. 1 (1.6-23.1)	0.008
BMI (kg/m^2^)								
≥18.5	183	9	1					
< 18.5	299	13	1.1 (0.5-2.7)	0.77				
Anemia, (%)								
No anemia	304	4	1		1		1	
Mild	186	10	3.8 (1.2-12.6)	0.03	2.2 (0.6-7.6)	0.21	2.6 (0.6-11.1)	0.19
Moderate and severe	114	20	16.0 (5.3-47.9)	< 0.001	6.5 (2.0-21.2)	0.002	10.9 (2.7-44.3)	0.001
History of IDU, (%)								
No	209	11	1					
Yes	357	21	1.1 (0.5-2.4)	0.76				
ART, (%)								
No	324	14	1					
Yes	287	20	1.7 (0.8-3.3)	0.12				

*Data were missing for gender (n = 1), CD4 (n = 10), BMI in 482 patients (n = 129), anemia status (n = 7), history of IDU (n = 45).

Sensitivity analysis: subjects with TB treatment within 3 months presentation excluded from analysis

Mild anemia: Hb 10.5 - 12.99 g/dL for men; and 10.5 - 11.99 g/dL for women, moderate-severe anemia: Hb < 10.5 g/dL.

ART: receive ART within 6 of presentation.

BMI, body mass index; IDU: injecting drug user; ART, anti retroviral; HR, hazard ratio;

### Iron status parameters in ART-naïve patients

Serum ferritin, sTfR, CRP and reticulocyte index were measured in a subgroup of 141 randomly selected patients. ART-naïve patients (n = 95) with available iron parameters had very high plasma ferritin concentrations (median 641 ng/mL, IQR: 242-1296), moderately elevated CRP concentrations (median 6 mg/mL, IQR: 2-34), low sTfR concentrations (median 1014 U/mL, IQR: 736-1322), and normal reticulocyte counts (median 0.8%, IQR: 0.6-1.5%).

**Figure 3:**
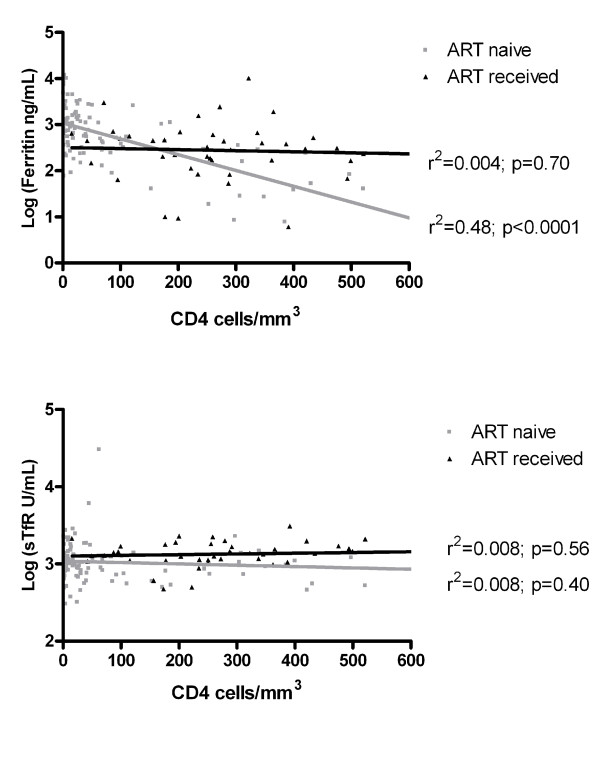
**Association between CD4 cell count and (a) Ferritin and (b) soluble Transferin receptor (sTfR) plasma concentrations in patients with (black) and without ART (grey)**. The lines are based on linear regression (n = 137).

Very high ferritin concentrations were found in subjects with CD4 counts below or equal to 50 cells/mm^3 ^(median 1078 mg/ml, IQR: 550-1824) compared to subjects with CD4 counts above 50 cells/mm^3 ^(median 279 mg/ml, IQR: 41-465, p < 0.001), and in subjects with moderate-severe anemia (median 1223 mg/ml, IQR: 519-2764) compared to subjects without (median 436 mg/ml, IQR: 245-1296. p = 0.07) or with mild anemia (median 532 mg/ml, IQR: 194-1099, p = 0.01). Moderately elevated hsCRP levels were also found in subjects with CD4 counts below or equal to 50 cells/mm^3 ^(median 12.2 mg/L, IQR: 2.0-63.2) compared to subjects with CD4 counts above 50 cells/mm^3 ^(median 4.9 mg/ml, IQR: 1.5-17.2, p = 0.08), and in subjects with moderate-severe anemia (median 18.4 mg/ml, IQR 4.5-94.4) compared to subjects without (median 2.0 mg/ml, IQR: 1.4-5.4, p = 0.001) or with mild anemia (median 6.0 mg/ml, IQR: 1.2-27.8, p = 0.03). Subjects with high ferritin, high hsCRP or low sTfR concentrations showed a higher mortality during follow-up, although this was not statistically significant (Table 3). Low ferritin levels (< 30 mg/ml) were found in 6.3% of patients, all were women with a CD4 cell count above 200 cells/mm^3^. There was a significant negative correlation between ferritin concentrations and CD4 count (Figure 3).

**Table 3:** Associations between iron and inflammatory parameters and mortality among ART-naïve patients (n = 95) in Hasan Sadikin Hospital, September 2007 - August 2009

			Single		Multiple*	
			
	n	mortality	HR (95% CI)	p value	HR (95% CI)	p value
Ferritin						
Low-normal	31	4	1		1	
High	64	16	1.8 (0.6-5.4)	0.29	1.3 (0.4-4.6)	0.68
sTfR						
Normal-high	10	4	1		1	
Low	85	16	2.2 (0.7-6.6)	0.16	1.7 (0.5-5.3)	0.38
hsCRP						
< 50 mg/L	41	5	1		1	
> 50 mg/L	54	15	2.2 (0.1-6.1)	0.12	2.1 (0.6-7.3)	0.24

*Cox proportional hazard models adjusted for CD4 cell count (< 50, > 50 cells/mm^3^), age (< 29, > 29 years old), anemia (no anemia Hb > 13 g/dl for men and > 12 g/dl for women, mild Hb < 10.5 - 12.99 g/dL for men; and < 10.5 - 11.99 g/dL for women, moderate-severe anemia: Hb < 10.5 g/dL), and gender.

Ferritin: High > 400 ng/mL for men or > 150 ng/mL for women, low-normal < 400 ng/mL for men or < 150 ng/mL for women; sTfR: Low < 1870 U/mL; normal-high > 1870 U/mL

HR, Hazard ration; CI: confidence interval; hsCRP, high sensitive C-reactive protein.

Predictors of hyperferritinemia in ART-naïve patients were subsequently determined in a univariate and multivariate model (Table 4). CD4 cell count below 50/mm^3 ^and hsCRP > 5 mg/L were associated with hyperferritinemia after correction for gender, degree of anemia, and HCV co-infection. After exclusion of patients with tuberculosis co-infection, low CD4 count was the only factor associated with hyperferritinemia.

**Table 4:** Multivariate analysis for factors related to high-ferritin concentrations among ART-naïve patients (n = 95) in Hasan Sadikin Hospital, September 2007 - August 2009

			Base analysis				Sensitivity analysis	
			
	n	high ferritin	Univariate		Multivariate		Multivariate	
			
			OR (95% CI)	p value	OR adjusted (95% CI)	p value	OR adjusted (95% CI)	p value
Gender								
Female	27	12	1		1		1	
Male	68	52	4.1 (1.6-10.4)	0.004	1.5 (0.5-5.1)	0.47	1.8 (0.50-6.2)	0.39
Anemia								
No anemia	15	10	1					
Mild	56	35	0.8 (0.3-2.8)	0.77				
Moderate - severe	24	19	1.9 (0.4-8.2)	0.39				
hsCRP (mg/L)								
< 5	41	21	1		1		1	
> 5	54	43	3.7 (1.5-9.2)	0.004	3.8 (1.3-10.9)	0.01	2.8 (0.9-8.4)	0.07
CD4 (cells/mL)								
> 50	33	12	1		1		1	
0-50	60	51	9.9 (3.6-27.0)	< 0.001	10.0 (3.5-28.8)	< 0.001	6.8 (2.2-20.3)	0.001
Anti HCV								
Negative	19	9	1					
Positive	36	25	2.5 (0.8-7.9)	0.11				

*CD4 cell counts were missing in 2 patients, HCV serostatus in 40 patients.

Sensitivity analysis: subjects with TB treatment within 3 months presentation excluded from analysis

High ferritin: > 400 ng/mL for men or > 150 ng/mL for women

Mild anemia: Hb 10.5 - 12.99 g/dL for men; and 10.5 - 11.99 g/dL for women, moderate-severe anemia: Hb < 10.5 g/dL.

ART, anti retroviral; OR, odd ratio; hsCRP, high sensitive C-reactive protein; HCV, hepatitis virus C.

### ART experienced patients

At time of enrollment in the cohort, 258 patients were already taking 1^st ^line ART, for a median of 24.0 months (range: 3.3 - 96.3 months). The majority of patients were on a ZDV containing regimens (62.8%), the others on a d4T containing regimen. Anemia was less common in patients taking ART: 14.3% had mild, 1.9% had moderate, and no patient had severe anemia (p < 0.001) (Table 1). The use of ZDV was associated with anemia: among patients taking ZDV, 20.3% had anemia, compared with 7.4% among those who were not taking ZDV (p = 0.01). During a median 19.8 months (IQR: 9.8-20.0), 2 (0.8%) ART experienced patients died and 26 (10.1%) were lost to follow-up.

Patients taking ART seemed to have lower plasma ferritin concentrations (median 304.1 ng/mL, IQR; 160.7-574.6) compared to ART-naïve patients, although this was not statistically significant (p = 0.12). In addition, they had lower hsCRP values (median 3.0 mg/mL, IQR: 0.9-6.3, p = 0.02), but similarly low sTfR concentrations (median 1378.5 U/mL, IQR: 1104.3-1798.5, p = 0.95) compared to ART-naïve patients. Duration of ART was not associated with plasma concentrations of ferritin or sTfR (data not shown). There was a significant negative correlation between ferritin concentrations and CD4 count in ART-naïve patients, but not in patients taking ART (Figure 3).

## Discussion

The results from our study indicate that anemia is highly prevalent among HIV patients in Indonesia and that moderate to severe anemia is strongly related to mortality. This is in line with findings from other studies [2,3,30-32]. We also report serum concentrations of ferritin and sTfR, both of which are often used for assessment of iron status. High serum ferritin concentrations were found, especially in patients with CD4 cell counts below 200 cells/mm^3^. Iron redistribution in the setting of a prolonged acute phase response is most likely responsible for this observation and this may explain the reported iron excess in the liver, bone marrow and other organs during the advanced stages of HIV [33]. Concentrations of CRP were however only moderately elevated and did not correlate well with ferritin concentrations. Although CRP is the most commonly used indicator of inflammation in daily practice, other acute phase response proteins such as a-1-acid-glycoprotein (AGP) may better reflect serum ferritin changes in inflammation because AGP remains elevated for a much longer time in sub-clinical chronic infections compared to CRP [15,34].

We found that sTfR concentrations were generally low and not related to CD4 count. This finding is in contrast to studies that showed that sTfR is not affected by HIV infection, even though these studies also report that sTfR alone has little value for differentiating anemia in the presence of inflammation [12,35,36]. We hypothesize that low sTfR concentrations more likely reflects the presence of some degree of bone marrow suppression by HIV. Together, these findings suggest that both serum ferritin and sTfR concentrations may not be reliable indicators of iron status in advanced HIV infection.

The prevalence of anemia was more than 40% in our untreated HIV-infected patients, more or less similar to studies from outside Indonesia [2,18,30,37]. Severe anemia was noticed in nearly 5% of our patients and this was higher compared to previous data from Europe [2] or Asia [3] but lower compared to a study from Africa [30]. Differences between the levels of immunodeficiency in the different studies may account for these differences. Our study also demonstrates that anemia is strongly related to increased mortality, as reported by others [2,3,30,31]. Interestingly, we found that this was also true after correction for CD4 cell count, indicating that anemia is an independent factor for HIV disease progression.

Measurement of iron parameters in a sub-group of patients revealed marked elevations in plasma ferritin, in line with earlier studies [14,17-19,35]. Our study shows that high ferritin levels are strongly (and inversely) related to CD4 cell numbers but not to gender, HCV co-infection nor CRP as a marker of inflammation, as has been suggested by others [19,37]. Apart from the prolonged acute phase response, the inverse relation between ferritin and CD4 cell count may also be caused by increased oxidative stress [38] related to depletion of CD4 cells [39]. Furthermore, The HIV virus itself may increase ferritin levels as HIV-1 Nef protein directly down-regulates the hemochromatosis protein HFE and as such causes iron accumulation [40]. However, the latter hypothesis was not supported by a study among HIV patients in Thailand, which reported no relation between serum ferritin concentrations and plasma HIV RNA [20]. Finally, the role of inflammation cannot be completely excluded since inflammatory pathways that do not include IL-6 and CRP can lead to high ferritin concentrations [41]. Interestingly, the inverse relation between ferritin levels and CD4 cell numbers disappeared after ART. This could be due to the effect of ART on oxidative stress, plasma HIV-RNA and/or inflammation. Our results argue against an important role of HCV as no relation was found between ferritin and HCV infection, although the number of subjects in whom all parameters were measured was limited.

Low ferritin levels (< 30 mg/ml) indicating iron deficiency were found in 6.3% of our patients and in 18.5% of the female patients. In previous studies from United States, iron deficiency anemia was found in 20% of HIV infected female injecting drug users [42], and iron supplementation was found to reduce anemia without adverse effects on HCV co-infection or plasma HIV-RNA [43]. However, iron supplementation should be done carefully and only in patients with iron deficiency as iron overload is associated with a poor prognosis of HIV-1 and hepatitis C virus infections and with growth of pathogenic microorganism [7]. Indeed, excess mortality was reported among HIV-infected patients receiving low-dose oral iron with dapsone for *Pneumocystis carinii *pneumonia prophylaxis [11]. In our study, we did not find a significant relation between plasma ferritin level and mortality, even though those who died on average had two-fold higher plasma ferritin levels.

Unlike ferritin, sTfR levels showed no association with CD4 cell counts, neither among ART-naïve nor among ART-experienced patients. Furthermore, sTfR levels were not different in anemic versus non-anemic subjects. Our sTfR levels seem slightly low, especially in ART-naïve patients suggesting that reduced erythropoiesis may contribute to the development of anemia, although no relation was found between sTfR levels and the presence or degree of anemia. Plasma sTfR concentrations were not associated with mortality during follow-up. This was also found in other studies which showed an increase of sTfR following ART [19,21], and no relation between sTfR levels and disease progression [19].

Our study has several limitations as it was observational, and as iron parameters were only measured in a subset of patients. Furthermore, no data are available on food or micronutrient intake which might affect iron status, and on occurrence of other opportunistic infections causing anemia. Tuberculosis could be important cause of anemia and could confound the results, although sensitivity analysis showed this was not the case. Furthermore we did not adjust ferritin concentrations for circulating HIV-RNA which are not measured routinely in ART-naïve patients. To be able to see the effects of ART on iron parameters, we excluded non-adherent patients. Loss to follow-up may have affected the estimated mortality, although we believe this did not affect our conclusions, since there was no significant difference in clinical characteristics including degree of anemia and CD4 cell count between patients with and without follow-up (data not shown).

## Conclusion

We can conclude that anemia, although usually mild, is highly prevalent in this setting and strongly related to immune-deficiency and mortality. Iron maldistribution and not iron deficiency seems to underlie the development of anemia while the role of compromised erythropoiesis and HCV co-infection seems limited.

## Competing interests

The authors declare that they have no competing interests.

## Authors' contributions

RW designed, performed statistical analysis and prepared the manuscript. RS, ARI and AZ, did laboratory examinations and help collected data. HJ, QdM, RvC and AvdV designed, gave advice on statistical analysis and prepared the manuscript. All authors read and approved the final manuscript.

## Pre-publication history

The pre-publication history for this paper can be accessed here:

http://www.biomedcentral.com/1471-2334/11/213/prepub
